# Supplementary material for: Comorbid anxiety and depression: Prevalence and associated factors among pregnant women in Arba Minch zuria district, Gamo zone, southern Ethiopia
Source: PLoS One. 2021 Mar 10;16(3):e0248331. doi: 10.1371/journal.pone.0248331 (PMC7946223; doi:10.1371/journal.pone.0248331)
Supplement: S1 Table — (DOCX) [file pone.0248331.s001.docx]

**Annex II: English Version Questionnaire**

| Name of the kebele: ________________________________ | | |
| --- | --- | --- |
| Code number of the questionnaire: ______________________________ | | |
| Interviewer’s name & signature: | Name:___________________ | Signature_________ |
| Supervisor’s name & signature: | Name :___________________ | Signature__________ |

| **Checklist used to determine the Pregnancy Status*** |  |
| --- | --- |
| **Questions** | **Response** |
| 1. Did your last menstrual period start within the last 7 day? | [1] Yes [2] No |
| 1. Have you had sex since your last menstrual period started? | [1] Yes [2] No |
| 1. Have you been using a reliable birth control method consistently and correctly since your last menstrual period started? | [1] Yes [2] No |
| 1. Did you have a miscarriage or abortion in the last 7 day? | [1] Yes [2] No |
| 1. Did you have a baby in the last 4 week? | [1] Yes [2] No |
| 1. Did you have a baby in the last 6 month?    1. Are you breastfeeding now?    2. Are your periods still gone since you had the baby? | [1] Yes [2] No  [1] Yes [2] No  [1] Yes [2] No |
| *Pregnancy was considered to be **unlikely** if the participant fulfilled any of these criteria: answered “Yes” to questions 1, 3, 4, or 5; answered “No” to question 2; or answered “Yes” to questions 6, 6a, and 6b. The health extension workers were instructed to pass the household if there is no pregnant woman after assessing using the above questions. | |

| **S/NO** | | **Question** | **Response** | | | | | | | | | | **Skip** | | | | |
| --- | --- | --- | --- | --- | --- | --- | --- | --- | --- | --- | --- | --- | --- | --- | --- | --- | --- |
| **Part I: Socio-demographic Characteristics** | | | | | | | | | | | | | | | | | |
|  | | How old are you? ( in completed years) | **______________** | | | | | | | | | |  | | | | |
|  | | What is your ethnicity? | 1. Gamo 2. Zeyisie 3. Oromo 4. Amhara 5. Other (specify)_______ | | | | | | | | | |  | | | | |
|  | | What is your religion? | 1. Protestant 2. Orthodox 3. Muslim 4. Other(specify)________ | | | | | | | | | |  | | | | |
|  | | What is your level of education? | 1. Unable to read and write 2. Able to read and write 3. Primary (1-8) 4. Attend grade 9-12 5. College and above | | | | | | | | | |  | | | | |
|  | | What is your husband’s level of education? | 1. Unable to read and write 2. Able to read and write 3. Attend Primary (1-8) 4. Attend grade 9-12 5. College and above | | | | | | | | | |  | | | | |
|  | | What is your marital status? | 1. Single 2. Married 3. Widowed 4. Divorced 5. Separated | | | | | | | | | |  | | | | |
|  | | What is your occupation? | 1. House wife 2. Farmer 3. Merchant 4. Government employee 5. Non-governmental employee 6. Others (specify)_______ | | | | | | | | | |  | | | | |
|  | | What is the main occupation of your husband? | 1. unemployed 2. Daily Laborer 3. Farmer 4. Trader/Merchant 5. Student 6. Employed 7. Other | | | | | | | | | |  | | | | |
|  | | What is your residence? | 1. Urban 2. Rural | | | | | | | | | |  | | | | |
|  | | How many persons live in your family (family size)? | __________ | | | | | | | | | |  | | | | |
|  | | What is your nearest health facility? | 1. Health post 2. Health center 3. Hospital | | | | | | | | | |  | | | | |
|  | | Distance from health facilities in KM | _____________ | | | | | | | | | |  | | | | |
| **Part II: Obstetrics Characteristics** | | | | | | | | | | | | | | | | | |
|  | | How old were you during your first marriage in completed years? | | | | _____________ | | | | | | | | |  | | |
|  | | Total number of pregnancy? | | | | _______ if 1 | | | | | | | | | **207** | | |
|  | | Number of live birth after viability? | | | | _________ | | | | | | | | |  | | |
|  | | Do you have history of stillbirth? | | | | 1. Yes 2. No | | | | | | | | |  | | |
|  | | Do you history of newborn death within the first 28 day’s | | | | 1. Yes 2. No | | | | | | | | |  | | |
|  | | Do you have history of abortion? | | | | 1. Yes 2. No | | | | | | | | |  | | |
|  | | When was the last menstruation? In D/M/Yr. | | | | ________________ | | | | | | | | |  | | |
|  | | Gestational age of the current pregnancy (in weeks) | | | | _______________ | | | | | | | | |  | | |
|  | | Does the current pregnancy wanted? | | | | 1. Yes 2. No | | | | | | | | |  | | |
|  | | Is your current pregnancy on scheduled time? | | | | 1. Yes 2. No | | | | | | | | |  | | |
|  | | Did you see anyone for antenatal checkup during the current pregnancy? | | | | 1. Yes 2. No | | | | | | | | | **214** | | |
|  | | Where did you attend your first Antenatal Checkup (ANC) visit? | | | | 1. Home 2. Health Post 3. Health center 4. Private Clinic 5. Hospital | | | | | | | | |  | | |
|  | | How many ANC visits did you attend for this pregnancy? | | | | _____________ | | | | | | | | |  | | |
|  | | During this pregnancy, did you experience any serious health problems related to the pregnancy? | | | | 1. Yes 2. No | | | | | | | | | **215** | | |
|  | | If yes What problems did you experience? | | | | | | | | | | | | |  | | |
|  | | Bleeding | | | | 1. Yes 2. No | | | | | | | | |  | | |
|  | | Swollen Hands/Face | | | | 1. Yes 2. No | | | | | | | | |  | | |
|  | | Blurred Vision | | | | 1. Yes 2. No | | | | | | | | |  | | |
|  | | Convulsions | | | | 1. Yes 2. No | | | | | | | | |  | | |
|  | | High Fever | | | | 1. Yes 2. No | | | | | | | | |  | | |
|  | | Loss of Consciousness | | | | 1. Yes 2. No | | | | | | | | |  | | |
|  | | Severe Abdominal Pain | | | | 1. Yes 2. No | | | | | | | | |  | | |
|  | | Water Breaks 12 hours before birth | | | | 1. Yes 2. No | | | | | | | | |  | | |
|  | | Discharge with unusual odor | | | | 1. Yes 2. No | | | | | | | | |  | | |
|  | | Pain during urination | | | | 1. Yes 2. No | | | | | | | | |  | | |
|  | | Severe Headache | | | | 1. Yes 2. No | | | | | | | | |  | | |
|  | | Severe weakness | | | | 1. Yes 2. No | | | | | | | | |  | | |
|  | | Other (Specify) ______________ | | | | 1. Yes 2. No | | | | | | | | |  | | |
|  | | Do you face any problem for the current pregnancy? | | | | 1. Yes 2. No | | | | | | | | | **301** | | |
|  | | If yes for Qn. 225. Where you go for help? | | | | 1. Traditional birth attendant 2. Traditional healer 3. Health institution 4. Religious places/holy water 5. Mental health professionals 6. Other specify____ | | | | | | | | |  | | |
|  | | Did you take any treatment for your problem? | | | | 1. Yes 2. No | | | | | | | | | **301** | | |
|  | | If yes does it helps you? | | | | 1. Did not help at all 2. Helped a bit 3. Helped a lot | | | | | | | | |  | | |
| **Part III: Interpersonal Relations**: No matter how well a couple gets along, there are times when they disagree. Couples get annoyed with the other person, or just have spats or fights because they’re in a bad mood or tired of for some other reason. They also use many different ways of trying to settle their differences. I’m going to ask what you and your partner might feel when you have an argument. | | | | | | | | | | | | | | | | | |
|  | | How do you and your partner work out arguments? | | 1. no difficulty 2. some difficulty 3. great difficulty | | | | | | | | | | | |  | |
|  | | In general, how do you describe your relationship? | | 1. no tension 2. some tension 3. a lot of tension | | | | | | | | | | | |  | |
|  | | How is your partner treating you and the kids? | | 1. always well 2. well most of the time 3. neutral 4. not well most of the time 5. never well | | | | | | | | | | | |  | |
|  | | Do you feel safe in your current relationship? | | 1. always safe 2. safe most of the time 3. neutral 4. not safe most of the time 5. never safe | | | | | | | | | | | |  | |
|  | | Considering your current partners, friends, or any past partners or friends, are there anyone who is making you feel unsafe now? | | 1. always safe 2. safe most of the time 3. neutral 4. not safe most of the time 5. never safe | | | | | | | | | | | |  | |
| **Part IV: Social support scale**: The following 3 questions ask about how you experience your social relationships. The inquiry is about your immediate personal experience. Please circle the option that represents your experience. | | | | | | | | | | | | | | | | | |
|  | | How many people are so close to you that you can count on them if you have serious personal problems? | | _____________________ | | | | | | | | | | | |  | |
|  | | How much concern do people show in what you are doing? | | 1. No concern and interest 2. Little concern and interest 3. Uncertain 4. Some concern and interest 5. A lot concern and interest | | | | | | | | | | | |  | |
|  | | How easy is it to get practical help from neighbors if you should need it? | | 1. Very difficult 2. Difficult 3. Possible 4. Easy 5. Very easy | | | | | | | | | | | |  | |
| **Part V: Experience of Threatening Events:** In the next questionnaire, I will read a list of potential unpleasant events you might have faced during the last 12 months. Please, respond “Yes” if you had faced the event in the past 12 months; “No” if not. | | | | | | | | | | | | | | | | | |
|  | |  | | | | | | | | **Yes** | | **No** | | | | |  |
|  | | In the last 6 months, have you yourself suffered a serious illness, injury or an assault? | | | | | | | | [1] | | [2] | | | | |  |
|  | | In the last 6 months has a serious illness, injury or assault happened to a close relative? | | | | | | | | [1] | | [2] | | | | |  |
|  | | In the last 6 months has your spouse, parent or child died? | | | | | | | | [1] | | [2] | | | | |  |
|  | | In the last 6 months has a close family friend or another relative died? | | | | | | | | [1] | | [2] | | | | |  |
|  | | In the last 6 months have you had a separation due to marital difficulties? | | | | | | | | [1] | | [2] | | | | |  |
|  | | In the last 6 months have you broken off a steady friendship or relationship? | | | | | | | | [1] | | [2] | | | | |  |
|  | | In the last 6 months have you had a serious problem with a close friend, neighbour or relative?) | | | | | | | | [1] | | [2] | | | | |  |
|  | | In the last 6 months have you had a major financial crisis (serious money worries)? | | | | | | | | [1] | | [2] | | | | |  |
|  | | In the last 6 months have you lost or had anything stolen which mattered lot you? | | | | | | | | [1] | | [2] | | | | |  |
|  | | In the last 6 months have you had any problems with the police or courts? | | | | | | | | [1] | | [2] | | | | |  |
|  | | In the last 6 months has your husband been not been able to work? | | | | | | | | [1] | | [2] | | | | |  |
|  | | In the past 6 months, have you had a physical fight with your spouse? | | | | | | | | [1] | | [2] | | | | |  |
| **Part VI: History of Substance Abuse** | | | | | | | | | | | | | | | | | |
|  | | How often do you have six or more drinks on one occasion? | | | 1. Never 2. once or less per month 3. Monthly 4. weekly 5. Daily | | | | | | | | |  | | | |
|  | | How often during the last year have you been unable to remember what happened the night before because you have been drinking? | | | 1. Never 2. once or less per month 3. Monthly 4. Weekly 5. Daily | | | | | | | | |  | | | |
|  | | How often during the last year have you failed to do what was normally expected of you because of drinking? | | | 1. Never 2. once or less per month 3. Monthly 4. Weekly 5. Daily | | | | | | | | |  | | | |
|  | | In the last year has a relative or friend or a doctor or other health worker been concerned about your drinking or suggested you cut down? | | | 1. Never 2. once or less per month 3. Monthly 4. Weekly 5. Daily | | | | | | | | |  | | | |
|  | | Did you have history of smoking in the last 12 months? | | | 1. Yes 2. No | | | | | | | | |  | | | |
|  | | Did you chew khat at least once in the last 12 months? | | | 1. Yes 2. No | | | | | | | | |  | | | |
| **Part VII: Medical History** | | | | | | | | | | | | | | | | | |
|  | | Have you ever admitted due to blood pressure in past pregnancy? | | | | | | 1. Yes 2. No | | | | | | | | |  |
|  | | Have you ever experienced fistula in past pregnancies? | | | | | | 1. Yes 2. No | | | | | | | | |  |
|  | | Are you currently suffering from diabetes? | | | | | | 1. Yes 2. No | | | | | | | | |  |
|  | | Are currently suffering from any renal disease? | | | | | | 1. Yes 2. No | | | | | | | | |  |
|  | | Are you currently suffering from any cardiac disease? | | | | | | 1. Yes 2. No | | | | | | | | |  |
|  | | Any other disease (ex TB, arthritis, HIV, Asthma, etc.)____ | | | | | | 1. Yes 2. No | | | | | | | | |  |
| **Part VIII: Depression assessment PHQ-9: I will ask you how often you experienced the following problems over the last 2 weeks. Your response could be often** (2-6 **days); several days** (7-11 **days); every day (**12-14 **days) depending on statements I read**. | | | | | | | | | | | | | | | | | |
| Items | | | | | | | **Not at all** | | **Several days** | | **Nearly half the days** | | | | | | **Every day** |
|  | Little interest or pleasure in doing things | | | | | | [1] | | [2] | | [3] | | | | | | [4] |
|  | Feeling down, depressed, or hopeless | | | | | | [1] | | [2] | | [3] | | | | | | [4] |
|  | Trouble falling or staying asleep, or sleeping too much | | | | | | [1] | | [2] | | [3] | | | | | | [4] |
|  | Feeling tired or having little energy | | | | | | [1] | | [2] | | [3] | | | | | | [4] |
|  | Poor appetite or overeating | | | | | | [1] | | [2] | | [3] | | | | | | [4] |
|  | Feeling bad about yourself - or that you are a failure or have let yourself or your family down | | | | | | [1] | | [2] | | [3] | | | | | | [4] |
|  | Trouble concentrating on things, such as reading the newspaper or watching television | | | | | | [1] | | [2] | | [3] | | | | | | [4] |
|  | Moving or speaking so slowly that other people could have noticed? Or the opposite - being so fidgety or restless that you have been moving around a lot more than usual | | | | | | [1] | | [2] | | [3] | | | | | | [4] |
|  | Thoughts that you would be better off dead or of hurting yourself in some way | | | | | | [1] | | [2] | | [3] | | | | | | [4] |

| **Part IX: Generalized anxiety disorder assessment tool (GAD 7): With in the last 2 weeks how often have you been bothered by any of the following problems:** | | | |
| --- | --- | --- | --- |
|  | Feeling nervous, anxious or on edge? | 1. Not at all 2. Several days 3. More than half the days 4. Nearly every day |  |
|  | Not being able to stop or control worrying? | 1. Not at all 2. Several days 3. More than half the days 4. Nearly every day |  |
|  | Worrying too much about different things? | 1. Not at all 2. Several days 3. More than half the days 4. Nearly every day |  |
|  | Trouble relaxing? | 1. Not at all 2. Several days 3. More than half the days 4. Nearly every day |  |
|  | Being so restless that it is hard to sit still? | 1. Not at all 2. Several days 3. More than half the days 4. Nearly every day |  |
|  | Becoming easily annoyed or irritable? | 1. Not at all 2. Several days 3. More than half the days 4. Nearly every day |  |
|  | Feeling afraid as if something awful might happen? | 1. Not at all 2. Several days 3. More than half the days 4. Nearly every day |  |

| **Part X: Questions to assess household food security condition (HFIAS): Rarely** =1X or 2X in the past four weeks), **Sometimes** = “3x to 10x in the past four weeks”, **Often**=”10x in the past four weeks” | | | |
| --- | --- | --- | --- |
| **Q. No** | **Questions** | **Response** | **skip** |
|  | In the past four weeks, did you worry that your household would not have enough food? | 1. Yes 2. No | 1002 |
| 1001a. | How often did this happen? | 1. Rarely 2. Sometimes 3. Often |  |
|  | In the past four weeks, were you or any household member not able to eat the kinds of foods you preferred because of a lack of resources? | 1. Yes 2. No | 1003 |
| 1002a | How often did this happen? | 1. Rarely 2. Sometimes 3. Often |  |
|  | In the past four weeks, did you or any household member have to eat a limited variety of foods due to a lack of resources? | 1. Yes 2. No | 1004 |
| 1003a | How often did this happen? | 1. Rarely 2. Sometimes 3. Often |  |
|  | In the past four weeks, did you or any household member have to eat some foods that you really did not want to eat because of a lack of resources to obtain other types of food? | 1. Yes 2. No | 1005 |
| 1004a | How often did this happen? | 1. Rarely 2. Sometimes 3. Often |  |
|  | In the past four weeks, did you or any household member have to eat a smaller meal than you felt you needed because there was not enough food? | 1. Yes 2. No | 1006 |
| 1005a | How often did this happen? | 1. Rarely 2. Sometimes 3. Often |  |
|  | In the past four weeks, did you or any other household member have to eat fewer meals in a day because there was not enough food? | 1. Yes 2. No | 1007 |
| 1006a | How often did this happen? | 1. Rarely 2. Sometimes 3. Often |  |
|  | In the past four weeks, was there ever no food to eat of any kind in your household because of lack of resources to get food? | 1. Yes 2. No | 1008 |
| 1007a | How often did this happen? | 1. Rarely 2. Sometimes 3. Often |  |
|  | In the past four weeks, did you or any household member go to sleep at night hungry because there was not enough food? | 1. Yes 2. No | 1009 |
| 1008a | How often did this happen? | 1. Rarely 2. Sometimes 3. Often |  |
|  | In the past four weeks, did you or any household member go a whole day and night without eating anything because there was not enough food | 1. Yes 2. No | Part XI |
| 1009a | How often did this happen? | 1. Rarely 2. Sometimes 3. Often |  |

| **Part XI: Questions to assess the current family wealth (economic condition)**  Could you tell me if you have the following in your house? | | |
| --- | --- | --- |
| **Asset type** | **Response** | |
| **Domestic animals** |  |  |
| Ox | No (0) | Yes (1) |
| Cow | No (0) | Yes (1) |
| Calf | No (0) | Yes (1) |
| Sheep | No (0) | Yes (1) |
| Goat | No (0) | Yes (1) |
| Horse | No (0) | Yes (1) |
| Donkey | No (0) | Yes (1) |
| Cock and Hen | No (0) | Yes (1) |
| **Durable assets** |  |  |
| Television | No (0) | Yes (1) |
| Radio | No (0) | Yes (1) |
| Electricity | No (0) | Yes (1) |
| Refrigerator | No (0) | Yes (1) |
| Conventional telephone | No (0) | Yes (1) |
| Mobile phone | No (0) | Yes (1) |
| Car | No (0) | Yes (1) |
| Motorcycle | No (0) | Yes (1) |
| Cycle | No (0) | Yes (1) |
| Cart | No (0) | Yes (1) |
| Gold, money | No (0) | Yes (1) |
| Ownership of owned living house | No (0) | Yes (1) |
| Ownership of agricultural land | No (0) | Yes (1) |
| **Productive assets** |  |  |
| Plough plow | No (0) | Yes (1) |
| Axe | No (0) | Yes (1) |
| Hoe | No (0) | Yes (1) |
| Shovel | No (0) | Yes (1) |
| Sickle | No (0) | Yes (1) |
| Modern beehive | No (0) | Yes (1) |
| Traditional beehive | No (0) | Yes (1) |
| **Housing characteristics** |  |  |
| Indoor plumping/ pipe water | No (0) | Yes (1) |
| Type of flooring | Earth/dung (0) | Cement/raw wood (1) |
| **Other household materials** |  |  |
| Bed | No (0) | Yes (1) |
| Table | No (0) | Yes (1) |
| Chair | No (0) | Yes (1) |
| Stove | No (0) | Yes (1) |

**I have finished thank you for your patience!!!**
