# Supplementary material for: Comorbid anxiety and depression: Prevalence and associated factors among pregnant women in Arba Minch zuria district, Gamo zone, southern Ethiopia
Source: PLoS One. 2021 Mar 10;16(3):e0248331. doi: 10.1371/journal.pone.0248331 (PMC7946223; doi:10.1371/journal.pone.0248331)
Supplement: S2 Table — (DOC) [file pone.0248331.s002.doc]

**S2 Table - STROBE Statement for cross-sectional studies**

|  | Item No | Recommendation |
| --- | --- | --- |
| **Title and abstract** | 1 | (*a*) Indicate the study’s design with a commonly used term in the title or the abstract  *The abstract method section describes that the study design is “cross-sectional study”.* |
|  |  | (*b*) Provide in the abstract an informative and balanced summary of what was done and what was found  *The abstract describes the methods and the key findings.* |
| Introduction | | |
| Background/rationale | 2 | Explain the scientific background and rationale for the investigation being reported  *The background and rationale of the study are explained under the introduction section, paragraphs 1-5.* |
| Objectives | 3 | State specific objectives, including any prespecified hypotheses.  *The purpose of the study are stated in the 5^th^ paragraph of the introduction* |
| Methods | | |
| Study design | 4 | Present key elements of study design early in the paper.  *The study design is described under the method section, paragraphs 1* |
| Setting | 5 | Describe the setting, locations, and relevant dates, including periods of recruitment, exposure, follow-up, and data collection.  *The study setting, study period and data collection are discussed in under the sub sections of the method ( paragraphs 1-11 of the Methods section)* |
| Participants | 6 | (*a*) Give the eligibility criteria, and the sources and methods of selection of participants.  *Eligibility criteria to be enrolled in the study is described in paragraph 2 of Methods section (under the study population subsection)* |
|  |  |  |
| Variables | 7 | Clearly define all outcomes, exposures, predictors, potential confounders, and effect modifiers. Give diagnostic criteria, if applicable.  *All variables are clearly discussed under the study variables and measurements subsection of the methods section (paragraphs 5-9 of the Methods section)* |
| Data sources/ measurement | 8* | For each variable of interest, give sources of data and details of methods of assessment (measurement). Describe comparability of assessment methods if there is more than one group.  *All variables are clearly discussed under the study variables and measurements subsection of the methods section (paragraphs 5-9 of the Methods section)* |
| Bias | 9 | Describe any efforts to address potential sources of bias.  *Exclusion criteria to described in paragraph 2, using validate tool to measure the of variables paragraphs 5-9 and use multivariable analysis to avoid cofounding paragraph 12 of the method section.* |
| Study size | 10 | Explain how the study size was arrived at  *Sample size determination for the study described in paragraph 3 of the Methods section* |
| Quantitative variables | 11 | Explain how quantitative variables were handled in the analyses. If applicable, describe which groupings were chosen and why  *Study variables are discussed in the study variables and measurements (paragraphs 5-9 of the Methods section) and Data Management and Analysis subsections (paragraph 12 of the method section)* |
| Statistical methods | 12 | (*a*) Describe all statistical methods, including those used to control for confounding *Statistical methods are discussed under data management and Analysis subsections (paragraph 12 of the method section)* |
|  |  | (*c*) Explain how missing data were addressed  *Not applicable* |
|  |  | (*d*) If applicable, explain how loss to follow-up was addressed  *Not applicable* |
|  |  | (*e*) Describe any sensitivity analyses  *Not applicable* |
| Results | | |
| Participants | 13* | (a) Report numbers of individuals at each stage of study—eg numbers potentially eligible, examined for eligibility, confirmed eligible, included in the study, completing follow-up, and analysed  *The number of participants involved in the study are discussed under the paragraph 1 of the result section (line one of the result section)* |
|  |  | (b) Give reasons for non-participation at each stage  *The number of participants involved in the study are discussed under the paragraph 1 of the result section (line one of the result section)* |
|  |  | (c) Consider use of a flow diagram  *Not applicable* |
| Descriptive data | 14* | (a) Give characteristics of study participants (eg demographic, clinical, social) and information on exposures and potential confounders  *Paragraph 1 of the result section*  *Frequency and percentages are reported in Table 1* |
|  |  | (b) Indicate number of participants with missing data for each variable of interest  *Not applicable* |
|  |  |  |
| Outcome data | 15* | Report numbers of outcome events or summary measures over time  *Proportion with 95% confidence interval is described in paragraph 3 of the result section* |
| Main results | 16 | (*a*) Give unadjusted estimates and, if applicable, confounder-adjusted estimates and their precision (eg, 95% confidence interval). Make clear which confounders were adjusted for and why they were included.  *The bivariate and multivariable results are presented in Table 3* |
|  |  | (*b*) Report category boundaries when continuous variables were categorized  *Not applicable* |
|  |  | (*c*) If relevant, consider translating estimates of relative risk into absolute risk for a meaningful time period  *Not applicable* |
| Other analyses | 17 | Report other analyses done—eg analyses of subgroups and interactions, and sensitivity analyses –  *Not applicable* |
| Discussion | | |
| Key results | 18 | Summarise key results with reference to study objectives  *Paragraph 1 of the Discussion section* |
| Limitations | 19 | Discuss limitations of the study, taking into account sources of potential bias or imprecision. Discuss both direction and magnitude of any potential bias  *Limitations are discussed in paragraph 8 of the Discussion section* |
| Interpretation | 20 | Give a cautious overall interpretation of results considering objectives, limitations, multiplicity of analyses, results from similar studies, and other relevant evidence *discussed in the discussion section paragraph 1-6* |
| Generalisability | 21 | Discuss the generalisability (external validity) of the study results  *Paragraph 7 of the Discussion section* |
| Other information | | |
| Funding | 22 | Give the source of funding and the role of the funders for the present study and, if applicable, for the original study on which the present article is based.  *Acknowledgments and Metadata* |

*Give information separately for exposed and unexposed groups.

**Note:** An Explanation and Elaboration article discusses each checklist item and gives methodological background and published examples of transparent reporting. The STROBE checklist is best used in conjunction with this article (freely available on the Web sites of PLoS Medicine at http://www.plosmedicine.org/, Annals of Internal Medicine at http://www.annals.org/, and Epidemiology at http://www.epidem.com/). Information on the STROBE Initiative is available at http://www.strobe-statement.org.
