# Supplementary material for: Comorbid anxiety and depression: Prevalence and associated factors among pregnant women in Arba Minch zuria district, Gamo zone, southern Ethiopia
Source: PLoS One. 2021 Mar 10;16(3):e0248331. doi: 10.1371/journal.pone.0248331 (PMC7946223; doi:10.1371/journal.pone.0248331)
Supplement: Attachment — Submitted filename: Reviewers comments PONE-D-20-08361R1.pdf [file pone.0248331.s004.pdf]

Reviewer's comments PONE-D-20-08361R1

Thank you for the opportunity to review this manuscript. This manuscript describes the prevalence of and factors associated with comorbid depression and anxiety during pregnancy in a community sample, in Ethiopia. Rates of both antenatal depression and anxiety have been shown to be concerningly high in other African studies, and both have negative consequences for mother and offspring. As the authors mention, there is a dearth of research on the comorbidity of these conditions, and therefore this is an important piece of work.

General

1. I feel that it would be beneficial to have this manuscript revised by a copy editor as there are numerous sentences which are confusing. An example:

Line 44- 45: *Depression is frequently occurs with anxiety and results an enormous adverse pregnancy and birth outcomes.*

Figures and tables

2. Please show all categories in Tables 1 and 2, and include a category for 'missing' data in instances where there is only data for some of the n=667 participants. Also, percentages should be calculated on the full analytic sample. For example, in Table 1, Husband's education should be displayed as follows:

No formal education	304	45.6
Primary education	222	33.3
Secondary education	87	13.0
College and above	37	5.5
Missing	*17*	*2.5*

3. In table 2, "*Status of current pregnancy*" could be called "reproductive intention"
4. Not convinced that Fig 2 is necessary.
5. Table 3: I find it confusing that the reference group is not listed first in this table – I have not seen results presented in this way before but could be mistaken.
6. I think it would be useful to see the p-value for the multivariate regression models presented in table 3.

Abstract

1. I do not feel that the first reviewer's concerns about the abstract have been addressed. There needs to be a stronger rationale for the current study, and the conclusion needs to speak to the results. The current conclusion reads as though a mindfulness intervention for improving mental health was undertaken alongside improving household food security.

Introduction

2. I do not feel that the prevalence of mental disorders in LMIC/ African antenatal populations was adequately discussed in the introduction, and noticed that there were African studies of mental health (including Ethiopian) which was not considered / cited. Authors cite Ethiopian studies in Line 66-68 – it would be interesting to know prevalence's found in these studies for context and comparison.

I realise that the literature on comorbid depression and anxiety is sparse, but there are some studies which may be useful:

- Sawyer A, Ayers S, Smith H. Pre- and postnatal psychological wellbeing in Africa: A systematic review. Journal of Affective Disorders 2010; 123(1–3): 17-29.
- Baron EC, Hanlon C, Mall S, et al. Maternal mental health in primary care in five low- and middle-income countries: a situational analysis. BMC health services research 2016; 16: 53.
- Redinger S, Pearson RM, Houle B, Norris SA, Rochat TJ. Antenatal depression and anxiety across pregnancy in urban South Africa. Journal of affective disorders 2020; 277: 296-305.
- Biaggi A, Conroy S, Pawlby S, Pariante CM. Identifying the women at risk of antenatal anxiety and depression: A systematic review. Journal of Affective Disorders 2016; 191: 62-77.
- (for medical condition comparison) Sowa NA, Cholera R, Pence BW, Gaynes BN. Perinatal depression in HIV-infected African women: a systematic review. *J Clin Psychiatry* 2015; **76**(10): 1385-96.

3. Line 45: Given the wide disparity in prevalences of depression and anxiety stated in the introduction, it would be help to have more information about the studies you cite when describing rates of comorbidity – for example were participants from HIC/LMIC or low/high risk pregnancies etc.

There is an increasing trend in comorbid anxiety and depression (CAD); Falah et.al. reported that 9.5% of 25,592 participants had self-reported 46 antenatal anxiety and depressive symptoms

4. Lines 50-55 explains the factors associated with "comorbid anxiety and depression" in the literature however only the bulk of cited literature is for either depression or anxiety. Might be useful to describe factors associated with one or the other in Africa/ Ethiopia specifically (as they are slightly different) and then tell the reader what is associated with comorbidity (this literature is sparse, might need to draw from HIC literature).

Methods

5. When using the PHQ-9 as a measure of probable depression, a cut-off score of 10 is what has been shown in validation studies to represent a depression, including in antenatal populations. I know that a cutoff of >5 has been used in Ethiopia before, and I assume the authors have based their analysis on this however the reasoning given by the authors in the manuscript is confusing and makes it seem like prevalence's are being under-estimated. I would reconsider this explanation. The same is true for the description of the GAD-7 cutoff and analysis.

Line score of ≥5 was used as a cut-off point for possible antenatal depression [23] due to small/null observations under moderate and moderately severe and severe depression.

Results

6. Lines 229 -231 Please state what the confounding factors in the model were?

7. Normal convention for reporting regression results: AOR 95% CI [CI-CI] p-value

8. The fact that women in the highest socioeconomic bracket had higher odds of having comorbid depression and anxiety warrants a more detailed explanation / discussion – especially since women reporting food insecurity were at higher risk. It would be interesting to look at/ mention what the unadjusted results were for this variable to see if they were in the same direction, or if there was collinearity with the food insecurity variable in the multivariate regression.

Discussion

9. Lines 260 and 261 – consider referencing.

10. Generally I echo the comments of the previous reviewer on the discussion section – that it is fairly written but would benefit from situating results within the LMIC then African then Ethiopian context.

11. Nice papers to look at when considering the results of food insecurity:

- Trujillo J, Vieira MC, Lepsch J, et al. A systematic review of the associations between maternal nutritional biomarkers and depression and/or anxiety during pregnancy and postpartum. *Journal of Affective Disorders* 2018; **232**: 185-203.
- *Abrahams Z, Lund C, Field S, Honikman S. Factors associated with household food insecurity and depression in pregnant South African women from a low socio-economic setting: a cross-sectional study. Social psychiatry and psychiatric epidemiology 2018; 53(4): 363-72.*
