# Supplementary material for: Comorbid anxiety and depression: Prevalence and associated factors among pregnant women in Arba Minch zuria district, Gamo zone, southern Ethiopia
Source: PLoS One. 2021 Mar 10;16(3):e0248331. doi: 10.1371/journal.pone.0248331 (PMC7946223; doi:10.1371/journal.pone.0248331)
Supplement: Attachment — Submitted filename: Response to Reviewers.docx [file pone.0248331.s003.docx]

**Rebuttal Letter**

September 13, 2020

Susan A. Bartels, MD, MPH, FRCPC
Academic Editor
PLOS ONE

**Rebuttal Letter:** Manuscript ID, PONE-D-20-08361

Dear Dr. Susan

Thank you for the opportunity to submit our manuscript “Comorbid anxiety and depression: prevalence and associated factors among pregnant women in Arba Minch zuria district, Gamo Zone, southern Ethiopia” for consideration for publication in PLOS ONE. We have carefully addressed the reviewers and editor comment in our responses and incorporated the related changes into the manuscript. To easily review our response: the comments and responses are written in black and dark blue font color respectively.

Thank you again for your consideration of this manuscript.

Sincerely,

Agegnehu Bante, MSc

Arba Minch University Ethiopia

**Editor’s Comment #1**

Line 15 - Suggest to avoid subjective and non-factual terms such as ‘earth-shattering’ in academic writing

Authors’ response: the introduction section of the abstract is revised and the term earth shattering is removed. Such errors are also identified in the rest of the document, amendments are done accordingly.

**Editor’s Comment #2**

Line 30 – in referring to the highest ranked quartile, please clarify if this means household income / socioeconomic level

Authors’ response: revised as ‘categorized in the highest socioeconomic level’

**Editor’s Comment #3**

Line 39 – While mental health during pregnancy is critically important, stating that it is ‘the major public health challenge in the world” might be contested particularly in light of the current global COVID-19 pandemic. Suggest to change tot “a major public health challenge”.

Authors’ response: since this study was conducted before covid-19 outbreak any misleading information like you mentioned are removed. The background section is modified including some citations and comments on this section are fully addressed.

**Editor’s Comment #4**

Line 42 – typo around topmost

Authors’ response: that statement was not specific to pregnant women, hence it is total removed.

**Editor’s Comment #5**

Line 43 – please clarify what a ‘pocket study’ is

Authors’ response: The phase was used just to indicate the study was conducted in one specific location (limited geographical area) of the country. However, to avoid any confusion for the readers the word pocket is removed in the revised file.

**Editor’s Comment #6**

Line 67 -  consider moving MBCT to the Discussion

Authors’ response: the statement is take to the discussion section and paraphrased with other mindfulness interventions

**Editor’s Comment #7**

Line 87 – ? rationale for excluding women who were on treatment for anxiety / depression

Authors’ response: The purpose of the study was to determine magnitude of comorbid anxiety and depression. Thus, excluding participants with confirmed mental health problem and initiate therapy underestimates the problem. Fortunately, there is no participant who was excluded by this criteria. Hence, the eligibility criteria to enroll in the study was revised as follow: All pregnant women who were living in Arba Minch Health and Demographic Surveillance Site (AM-HDSS) were the study population. Women were enrolled in to the study irrespective of their age and pregnancy trimester. Those pregnant women who were not able to communicate due to serious illness and not available after three consecutive home visits were excluded from the study.

**Editor’s Comment #8**

Line 104 – what are pregnancy screen checks and how where they used in the sampling?

Authors’ response: The first step in the sampling procedure was identifying pregnant women in the selected cluster. The Arba Minch Health and Demographic Surveillance Site data base and the health extension workers registry was used to identify pregnant women. In addition, the WHO and CDC pregnancy assessment checklist; which is designed to determine whether the woman is pregnant or not just for family planning service, was used to reasonable sure the woman is pregnant. The detail of the questions and how they used are included under the data collection tool and uploaded as a supplementary file.

**Editor’s Comment #9**

Line 128 – how many participants completed the pilot? It would be preferable to simply state the # rather than indicate 5% of the population

Authors’ response: the minimum sample required for this study was calculated as 654. However, due to the nature of the cluster sampling a total of 676 pregnant women were identified in the selected cluster. Hence, 5% of 676; 34 pregnant women who lived in Arba Minch town were selected for the pretest and amendment was done based on the result.

**Editor’s Comment #10**

Line 162 – Ethics section could use more details such as whether compensation was provided, how exactly referrals for were done, if a participant was suicidal how was that managed?

Authors’ response: participation in the current study was fully voluntary and there is no compensation that directly given for the participant. Since, all participants were under the Arba Minch health demographic surveillance site those who were screened positive for depression/anxiety were easily identified by their household number in the database. In general the ethics statement is revised as follow: Ethical approval for the study protocol was provided by Arba Minch University, college of medicine and health sciences, Institutional Review Board (IRB) with an ethical clearance number of CMHS/12031653/111. Moreover, this study was conducted under the Declaration of Helsinki, and the participants were informed of the purpose of the study. Furthermore, before the commencement of data collection written informed consent was obtained from all participants. To maintain the confidentiality of information gathered from each study participant, code numbers were used throughout the study. Pregnant women who screed positive for depression or anxiety received onsite mindfulness interventions. In addition, they were linked with the health extension workers for regularly psychological interventions and referral to health facilities in case of sever case like suicidal ideation.

**Editor’s Comment #11**

Line 182 – the article would benefit from close proof reading for typos, example line 182 “got pregnant” rather than “get pregnant”

Authors’ response: the manuscript passes through careful proof reading by all authors; Grammar and editorial issues including the raised one are addressed

**Editor’s Comment #12**

Results – Suggest to collapse some of the descriptive tables, not necessary to have a separate table for each major variable

Authors’ response: the result section is presented shortly; some extra details are removed and presented only in 3 tables and 3 figures with some text paraphrasing.

**Editor’s Comment #13**

Line 259 – explanation around the association between CAD and income status is not clear as written

Authors’ response: This might be because of economic status increased the need for the individual also increased and struggle to acquire more capital that leads to mental disorder. Also, in some cultures everything is controlled by the husband, the mother does nothing without the permission of her partner. Furthermore, as the wealth of the family improved the husband needs an extra wife, which is commonly exercised during pregnancy.

**Editor’s Comment #14**

Line 263 – does this mean that men take an additional wife when their first or earlier wives are pregnant?

Authors’ response: the interpretation is revised as it placed in the preceding comment. But let me explain your concern:

Although it is diminished, the culture allows two and more wives if the man is rich and able to fulfill the need of all his wives. However, in realty there is a conflict of interest between wives and the husband lives here and there such norms put tremendous impact in women.

**Editor’s Comment #15**

Discussion – as currently written the Discussion is mostly a compare / contrast of the results with other existing studies. It would be improved by going deeper into what the results mean for women in Ethiopia, what policies/programs/services are in place for pregnant women with CAD, what should be in place, what are additional areas for future research, etc.?

Authors’ response: all the ideas raised here are added in the last two paragraphs of the discussion as follow: The present study revealed that CAD in pregnant women is common in the study area. The study provides invaluable information for healthcare providers to address potential risk factors for depression and anxiety. Despite prenatal CAD results in serious health consequence both for the mother and her fetus, mental health services are very poor in Ethiopia. Scholars suggested that mindfulness-based cognitive therapy (MBCT) has imperative to decrease depression and anxiety in pregnant women. However, in Ethiopia, guidelines/manuals used to treat pregnant women with depression/anxiety are not available and the healthcare providers working in the antenatal care units are not able to screen and manage these conditions. In low income countries like Ethiopia, limited mental health centers and very low psychiatry professional, healthcare providers working in the antenatal care unit must be trained about common mental disorders screening and mindfulness interventions given for pregnant women. Moreover, maternal mental health must be integrated with other services like reproductive health, maternal and child health care. Psychiatry professional in collaboration with the federal ministry health should develop maternal mental health intervention guidelines.

Even though this study follows scientific methodology, it might encounter the following limitations: social desirability bias might underestimate the response of some measurements such as intimate partner violence substance abuse, household food insecurity. Moreover, the GAD-7 used to measure anxiety was not validated in Ethiopian context; which might affect the result of the current study. Thus, researchers and psychiatry professional must develop locally validated mental disorder screening tools.

**Editor’s Comment #16**

The authors are also encouraged to use a cross-sectional study check list (ex. STROBE) to ensure that standards for reporting are met.

Authors’ response: the STORBE-checklist is filled and uploaded as a supplementary information in the system

**Editor’s Comment #17**

Please ensure that your manuscript meets PLOS ONE's style requirements, including those for file naming. The PLOS ONE style templates can be found at

[https://journals.plos.org/plosone/s/file?id=wjVg/PLOSOne_formatting_sample_main_body.pdf](https://journals.plos.org/plosone/s/file?id=wjVg/PLOSOne_formatting_sample_main_body.pdf%20) &<https://journals.plos.org/plosone/s/file?id=ba62/PLOSOne_formatting_sample_title_authors_affiliations.pdf>

Authors’ response: the revised manuscript meets all the journal style requirements;

**Editor’s Comment #18**

2. In the ethics statement in the Methods and online submission information, please clarify whether consent was written or verbal.  If verbal, please also specify: 1) whether the ethics committee approved the verbal consent procedure, 2) why written consent could not be obtained, and 3) how verbal consent was recorded. If your study included minors, state whether you obtained consent from parents or guardians. If the need for consent or parental consent was waived by the ethics committee, please include this information.

Authors’ response: the consent was written and it is modified both in the method section and online submission. Let us explain more about the ethical statement:

The participant information sheet and informed voluntary consent was prepared in English and Amharic language. The information sheet mainly includes the title, purpose of the study, procedure and duration, risk and benefits, confidentiality, rights and contact address for any query. In addition, there is declaration of informed voluntary consent statement that was given for the participant for signature; it states that *“I have read/was read to me the participant information sheet. I have clearly understood the purpose of the study, the procedures, the risks and benefits, issues of confidentiality, the rights of participating and contact address for any queries. I have been given the opportunity to ask questions for things that may have been unclear. I was informed that I have the right to withdraw from the study at any time or not to answer any question that I do not want. Therefore, I declare my voluntary consent to participate in this study with my initials (signature).”* Furthermore, the proposal received an ethical clearance letter from Arba Minch University, college of medicine and health sciences, Institutional Review Board (IRB) with an ethical clearance number of CMHS/12031653/111.

**Reviewers comment**

Reviewer #1: Thank you very much for the opportunity to review this manuscript. Although the manuscript focused on an important area of research in Ethiopia, there are several issues which need to be addressed by the authors. Below are the concerns:

General comments:

**Reviewer #1 comment #1**

1. There are so many grammatical errors throughout the manuscript. Language editing is recommended.

Authors’ response: as mentioned above the manuscript pass through proof reading by all authors for many times. In addition, grammarly free application was used to avoid some spelling and grammar errors.

**Reviewer #1 comment #2**

2. There are too many unscientific language use throughout the manuscript. For instance, “Despite anxiety and depression have an earth-shattering impact…”.

Authors’ response: all the authors go through the whole document and replace unscientific/ unclear words with simple and easily understandable words.
ABSTRACT:

**Reviewer #1 comment #3**
1. The background needs to be re-written to clearly outline the rational for the present study.

Authors’ response: based on the suggestion the background section is revised by incorporating more recent articles and the rational of the study is explained by showing the research gap. Even though the prevalence of depression in pregnant women is studied, as far as our knowledge studies that determines the prevalence of comorbid depression and anxiety; anxiety alone are not researched. Hence, to design comprehensive mental disorder interventions the current study has paramount contribution. In the revise manuscript the rational of the study is incorporated.

**Reviewer #1 comment #4**

2. The conclusion is too broad. It needs to be focused on the key findings from the study.

Authors’ response: the conclusion written based on the key findings and revised as: In general, one in every ten women were affected by CAD. Early identification and treatment of pregnancy dangers signs and medical illness, incorporating mindfulness interventions as part of the routine antenatal care and improving household food security have paramount importance to minimize prenatal mental health problems.

INTRODUCTION

**Reviewer #1 comment #5**

1. The rationale for the present study is not clearly stated. The authors cited a study in Ethiopia by Woldetsadik et al. (2019) on prenatal mental health problems. How different is the current study in terms of added value. These issues should be clearly stated.

Authors’ response: The study conducted by Woldetsadik et al. (2019) in Bale zone South-East Ethiopia mainly focused on depression alone despite the running title is about *“Prevalence of common mental disorder and associated factors among pregnant women”*. In addition the measurement scale used to assess depression and anxiety is totally different. In our study the patient health questioner (PHQ); which has good internal consistency and validated in Ethiopian context was used for the antenatal depression assessment. Likewise, antenatal anxiety was assessed using the Generalised Anxiety Disorder (GAD-7); which is a useful and a validated tool to assess perinatal anxiety. On the contrary, the study conducted by Woldetsadik et al. (2019) used the WHO Self-Reported Questionnaire (SRQ); which is mainly used to assess depression. The paper was cited just to show the previous studies conducted in Ethiopia are manly depression alone. Based on the suggestion, the citation is removed from the background and discussion section it is not possible to compare the depression alone with that of co-morbid depression and anxiety.

**Reviewer #1 comment #6**

2. The authors need to provide more information on the choice of potential risk and protective factors by stating the theoretical bases for these factors.

Authors’ response: after extensive review of literature all variables associated with the co-morbid anxiety and depression. In the revised version these variables are in a separate paragraph. In the main document the conceptual framework and the relationship of each variable was clearly explained. However, presenting the theoretical bases for each variable make the background bulky and too long. Therefore, factors associated with CAD are presented in short way.

**Reviewer #1 comment #7**

3. Lines 39 & 40. The statements need to be edited and referenced as the authors are making factual claims.

Authors’ response: as per the suggestion; the statement is rewritten and cited. In addition, some of the statement are replaced with more recent data.
METHODS

**Reviewer #1 comment #8**

1. Exclusion and inclusion criteria need to be clearly spelt out.

Authors’ response: excluding pregnant women who initiate treatment for mental health problems underestimates the magnitude of the problem and in the revised manuscript the eligibility criteria for the study is modified as: All pregnant women who were living in Arba Minch Health and Demographic Surveillance Site (AM-HDSS) were the study population. Women were enrolled in to the study irrespective of their age and pregnancy trimester. Those pregnant women who were not able to communicate due to serious illness and not available after three consecutive home visits were excluded from the study.

**Reviewer #1 comment #9**

2. Under “Sampling procedure”, the authors need to provide further details on “pregnancy screening checklist”.

Authors’ response: the first step of the data assortment process was identified pregnant women from the selected clusters. Thus, the Arba Minch Health and Demographic Surveillance and the health extension workers pregnancy register were used to identify pregnant women in each cluster. In addition, to minimize the chance of missing early pregnancies the WHO and CDC pregnancy screening checklist was used to reasonable determine the pregnancy status of the woman (Table 1).

Table 1: Checklist used to determine the Pregnancy Status of women in Arba Minch zuria, Gamo zone, southern Ethiopia, 2019

| **Questions** | **Response** |
| --- | --- |
| 1. Did your last menstrual period start within the last 7 day? | [1] Yes [2] No |
| 2. Have you had sex since your last menstrual period started? | [1] Yes [2] No |
| 3. Have you been using a reliable birth control method consistently and correctly since your last menstrual period started? | [1] Yes [2] No |
| 4. Did you have a miscarriage or abortion in the last 7 day? | [1] Yes [2] No |
| 5. Did you have a baby in the last 4 week? | [1] Yes [2] No |
| 6. Did you have a baby in the last 6 month?    a. Are you breastfeeding now?    b. Are your periods still gone since you had the baby? | [1] Yes [2] No  [1] Yes [2] No  [1] Yes [2] No |
| *Pregnancy was considered to be **unlikely** if the participant fulfilled any of these criteria: answered “Yes” to questions 1, 3, 4, or 5; answered “no” to question 2; or answered “Yes” to questions 6, 6a, and 6b. The health extension workers were instructed to pass the household if there is no pregnant woman after assessing using the above questions. | |

**Reviewer #1 comment #10**

3. The sections “Data collection tool” and “Study variables” should be put together. The authors need to provide detailed information on each study measure in terms of developers, number of items, response format and scoring as well as cut-offs.

Authors’ response: the study variables section is merged to the “data collection tool” section and the detail of each variables measurement are described as follow:

**Outcome variables measurement scales and their measurement**

**Depression:** The Patient Health Questionnaire (PHQ-9) is originally designed by Doctors Spitzer, Williams and Kroenke was used for depression assessment. PHQ-9 score range from 0 to 27, since each of the 9 items can be scored from 0 (not at all) to 3 (nearly every day) [1]. Based on this severity of can be categorized as: 0-4 none, 5-9 mild, 10-14 moderate, 15-19 moderately severe, 20-27 severe. In our case, depression was categorized in to two; a score of ≥ 5 was used as a cutoff point to categorize pregnant women as having depression because there were small observation under moderate (13 observations) and moderately severe (6 observations) depression category and no observation under severe depression. Hence, the mild, moderate and moderately severe depression were merged as one category and labeled as “depression” [2].

**Anxiety:** The Generalized Anxiety Disorder 7-item (GAD-7) scale, developed by Robert L. Spitzer, MD, served as professor of psychiatry at Columbia University for 49 years, was used for anxiety assessment. GAD-7 is a valid and efficient tool for screening for generalized anxiety disorder in clinical practice and research [3]. GAD-7 score range from 0 to 21, since each of the 7 items can be scored from 0 (not at all) to 3 (nearly every day). Scores of 5, 10, and 15 are taken as the cut-off points for mild, moderate and severe anxiety, respectively. In our study score of ≥5 was taken as the cutoff point to categorize pregnant women as having anxiety [4]; there is no observation with a score of ≥15 (sever) and only 19 observation with a score of 10-14 (moderate).

**Independent variables and measurements (i.e. composite variables)**

**Substance abuse:** it was assessed using Fast Alcohol Screening Test (FAST) scale developed from the Alcohol Use Disorders Identification Test (AUDIT) by Hodgson et al. 2002. FAST has 4 item with a score ranges from 0 to 16, where a score of ≥3 indicates harmful drinking [5].

**Intimate partner violence (IPV):** it was assessed using five item; Women's Abuse Screening Test (WAST) originally developed by Brown et al. 1996 [6] was used. WAST score ranges from 0-16 where a score > 1 indicates the presence of violence [7].

**Social Support:** Oslo Social Support Scale (OSSS-3) which has three item scale and originally developed in Neverland’s was used. Its score ranges from 3-14 and categorized into three as: “poor social support” 3-8, “moderate support” 9-11 and “strong support” 12-14 [8].

**Life events:** The List of Threatening Experiences (LTE) developed by Brown & Harris (1978) was used; LTE is self-reported questionnaire with a list of 12 threatening events that invite a dichotomous response (yes/no). The number of life events were counted for each respondent and recorded as a value ranging from 0 to 12 [9]. LTE has good test-retest reliability and internal
consistency and widely used across the globe.

**Food security status:** it was measured using the Household Food Insecurity Access Scale (HFIAS) **developed** by **Food** and Nutrition Technical Assistance (FANTA) Program of the U.S. Agency for International Development. It has 9 items with a follow-up question about the frequency of occurrence if the response is yes for the main questions. The score range from 0 to 27. The HFIAS category was calculated for each household using the following command:

**Food Secure:** HFIA category = 1 if [(Q1a=0 or Q1a=1) and Q2=0 and Q3=0 and Q4=0 and Q5=0 and Q6=0 and Q7=0 and Q8=0 and Q9=0]

**Mildly Food Insecure Access:** HFIA category = 2 if [(Q1a=2 or Q1a=3 or Q2a=1 or Q2a=2 or Q2a=3 or Q3a=1 or Q4a=1) and Q5=0 and Q6=0 and Q7=0 and Q8=0 and Q9=0]

**Moderately Food Insecure Access:** HFIA category = 3 if [(Q3a=2 or Q3a=3 or Q4a=2 or Q4a=3 or Q5a=1 or Q5a=2 or Q6a=1 or Q6a=2) and Q7=0 and Q8=0 and Q9=0]

**Severely Food Insecure Access:** HFIA category = 4 if [Q5a=3 or Q6a=3 or Q7a=1 or Q7a=2 or Q7a=3 or Q8a=1 or Q8a=2 or Q8a=3 or Q9a=1 or Q9a=2 or Q9a=3]

In the current study, for the multivariable analysis household food security was categorized in to two as “food secure” when the participants didn’t experience food insecurity (access) conditions in the past 4 weeks and “food insecure” when the unable to access sufficient food at all time to lead active and healthy life [10].

**Reviewer #1 comment #11**

4. The reliability values of the study instruments in the current study need to be stated.

Authors’ response: the reliability of the PHQ-9 and GAD-7 were calculated for this particular study and the Cronbach’s alpha for the PHQ-9 = 87 and for the GAD-7=0.83

Table 2: Stata output for the reliability values for the PHQ-9 and GAD-7

| PHQ-9 | GAD-7 |
| --- | --- |
| Command  alpha ltlintrst-ththurt  **Output**  Test scale = mean(unstandardized items)  Average interitem covariance: .1030973  Number of items in the scale: 9  Scale reliability coefficient: **0.8676** | Command  alpha flntvos-afraid  **Output**  Test scale = mean(unstandardized items)  Average interitem covariance: .1175975  Number of items in the scale: 7  Scale reliability coefficient: **0.8322** |

**Reviewer #1 comment #12**

5. The authors used “mindful interventions” throughout the manuscript. This needs to be corrected as it is “mindfulness interventions”.

Authors’ response: checked throughout the document and amendment was done accordingly

**Reviewer #1 comment #13**

6. Ethical clearance number needs to be provided if available.
Authors’ response: yes it is available, “CMHS/12031653/111”, and incorporated in the revised manuscript
RESULTS

**Reviewer #1 comment #14**

1. Table 2. Education category “uneducated” should be changed to “no formal education”.

Authors’ response: “uneducated” replaced with “No formal education”

**Reviewer #1 comment #15**

2. The results need to be summarised as the presentation of frequencies and percentages of each predictor variables makes the results to lengthy. I would suggest three key tables (Socio-demographic, Chi-Square and Logistic regression). You have the cut-offs or categorizations of each of these variables and therefore, there is no need for presenting all the items.

Authors’ response: some of the table are removed only the socio-demographic, obstetrics and the logistic regression table are presented. The chi-square is already incorporated under the logistic regression hence no need of presenting it separately.

**Reviewer #1 comment #16**

3. Check the spelling of ANXIETY in Fig 4.
Authors’ response: yes it is checked and revised; the revised figure is uploaded to the system
DISCUSSION

**Reviewer #1 comment #17**
1. The discussion is fairly written. However, results need to be situated in the Ethiopian context and not merely comparing with previous studies.

Authors’ response: as mentioned under the editor’s comment section the discussion mainly shows the existing interventions for maternal mental disorders and forwards future direction both for researchers, psychiatry professional and other concerned stakeholders: key points added under the discussion section are presented as follow:

Despite prenatal CAD results in serious health consequence both for the mother and her fetus, mental health services are very poor in Ethiopia. Scholars suggested that mindfulness-based cognitive therapy (MBCT) has imperative to decrease depression and anxiety in pregnant women. However, in Ethiopia, guidelines/manuals used to treat pregnant women with depression/anxiety are not available and the healthcare providers working in the antenatal care units are not able to screen and manage these conditions. In low income countries like Ethiopia, limited mental health centers and very low psychiatry professional, healthcare providers working in the antenatal care unit must be trained about common mental disorders screening and mindfulness interventions given for pregnant women. Moreover, maternal mental health must be integrated with other services like reproductive health, maternal and child health care. Psychiatry professional in collaboration with the federal ministry health should develop maternal mental health intervention guidelines.

**Reviewer #2 comment**

I think it is novel idea in antenatal women who have comorbid anxiety and depression which is public health challenge in low and middle income country. The paper is an important one that shows predictors of comorbid anxiety and depression, economical factors, life events that encounter pregnant women, house hold food insecurity. One of the strength of this study also it community base study that taken from demographic surveillance site of Arba Minch university.
Authors’ response: Thank You!!

**References**

1.	Kroenke, K., R.L. Spitzer, and J.B.J.J.o.g.i.m. Williams, *The PHQ‐9: validity of a brief depression severity measure.* 2001. **16**(9): p. 606-613.

2.	Bitew, T., et al., *Antenatal depressive symptoms and maternal health care utilisation: a population-based study of pregnant women in Ethiopia.* BMC Pregnancy Childbirth, 2016. **16**(1): p. 301.

3.	Spitzer, R.L., et al., *A brief measure for assessing generalized anxiety disorder: the GAD-7.* 2006. **166**(10): p. 1092-1097.

4.	Zhong, Q.-Y., et al., *Diagnostic validity of the Generalized Anxiety Disorder-7 (GAD-7) among pregnant women.* PloS one, 2015. **10**(4).

5.	Hodgson, R., et al., *The FAST alcohol screening test.* 2002. **37**(1): p. 61-66.

6.	Brown, J.B., et al., *Development of the Woman Abuse Screening Tool for use in family practice.* 1996. **28**: p. 422-428.

7.	Rabin, R., et al., *Intimate partner violence screening tools.* American Journal of Prev Med, 2009. **36**(5): p. 439.

8.	Boen, H., *Characteristics of senior centre users – and the impact of a group programme on social support and late-life depression.* Norsk Epidemiologi 2012. **22**(2): p. 261.

9.	Brugha, T. and D. Cragg, *The List of Threatening Experiences: the reliability and validity of a brief life events questionnaire.* Acta Psychiatr Scand, 1990. **82**(1): p. 77.

10.	Coates, J., A. Swindale, and P. Bilinsky, *Household Food Insecurity Access Scale (HFIAS) for measurement of food access: indicator guide. Washington, DC: food and nutrition technical assistance project, academy for educational Development, 34.* 2007.
