# Supplementary material for: Comorbid anxiety and depression: Prevalence and associated factors among pregnant women in Arba Minch zuria district, Gamo zone, southern Ethiopia
Source: PLoS One. 2021 Mar 10;16(3):e0248331. doi: 10.1371/journal.pone.0248331 (PMC7946223; doi:10.1371/journal.pone.0248331)
Supplement: Attachment — Submitted filename: Response to Reviewers.docx [file pone.0248331.s005.docx]

**Rebuttal Letter**

February 10, 2021

Susan A. Bartels, MD, MPH, FRCPC
Academic Editor
PLOS ONE

**Rebuttal Letter:** Manuscript ID, PONE-D-20-08361

Dear Dr. Susan

Thank you for the opportunity to submit our manuscript “Comorbid anxiety and depression: prevalence and associated factors among pregnant women in Arba Minch zuria district, Gamo Zone, southern Ethiopia” for consideration for publication in PLOS ONE. We have carefully addressed the reviewers' and editor's comments in our responses and incorporated the related changes into the manuscript. To easily review our response: the comments and responses are written in black and dark blue font color respectively.

Thank you again for your consideration of this manuscript.

Sincerely,

Agegnehu Bante, MSc in maternity and neonatal nursing

Arba Minch University, Ethiopia

**Reviewer's comments**

**Reviewer #3**

Thank you for the opportunity to review this manuscript. This manuscript describes the prevalence of and factors associated with comorbid depression and anxiety during pregnancy in a community sample, in Ethiopia. Rates of both antenatal depression and anxiety have been shown to be concerningly high in other African studies, and both have negative consequences for mother and offspring. As the authors mention, there is a dearth of research on the comorbidity of these conditions, and therefore this is an important piece of work.

**General**

1. I feel that it would be beneficial to have this manuscript revised by a copy editor as there are numerous sentences which are confusing. An example:

Line 44- 45: *Depression is frequently occurs with anxiety and results an enormous adverse pregnancy and birth outcomes.*

**Authors’ response:** after addressing all the constructive comments you raised, the final version of the manuscript go through proof reads by the corresponding author, co-authors and senior scholars. Free online article paraphrasing too, plagiarism checker and grammarly was used for grammatical and spelling errors. Besides, the manuscript is written in simple and easily understandable English. Thus, in the current version all misleading, and unclear information is written understandably.

2. **Figures and tables**

Please show all categories in Tables 1 and 2, and include a category for ‘missing’ data in instances where there is only data for some of the n=667 participants. Also, percentages should be calculated on the full analytic sample. For example, in Table 1, Husband’s education should be displayed as follows:

| No formal education | 304 | 45.6 |
| --- | --- | --- |
| Primary education | 222 | 33.3 |
| Secondary education | 87 | 13.0 |
| College and above | 37 | 5.5 |
| *Missing* | *17* | *2.5* |

**Authors’ response**: In our case, it is not missing rather skip questions. For example, in table 1 ‘husband education’ and ‘husband occupation’ were not asked from single, divorced and widowed mothers that is why the denominator is 650 and the valid percentage is calculated based on this value. Likewise, in Table 2: the percentage for ‘history of stillbirth’, history of neonatal death, history of abortion’ were calculated by taking the denominator 546 because primigravida mothers were not asked these questions. Furthermore, “the number of ANC visits and Place of ANC follow-up” were asked only from those who already initiate ANC follow-up (i.e. 380 participants). In general, the reads can easily understand the source of these missing’s; hence, your suggestion is incorporated in the revised manuscript both in table 1& 2.

3. In table 2, “*Status of current pregnancy”* could be called “reproductive intention”

**Authors’ response:** Amended as recommended

Not convinced that Fig 2 is necessary.

**Authors’ response:** based on your recommendation after evaluating its importance figure 2 is removed from the revised manuscript because it does not give any additional benefit for the manuscript as well as for the readers.

4. Table 3: I find it confusing that the reference group is not listed first in this table – I have not seen results presented in this way before but could be mistaken. I think it would be useful to see the p-value for the multivariate regression models presented in table 3.

**Authors’ response:** thank you for your concern. Regarding the first concern; as far as I know, there is no standard to put the reference category at first or last. However, the selection of the reference category is based on the proportion of the outcome of interest (i.e. based on the cross-tabulation proportions; better to consider the variable with low proportion as a reference category). Thus, during the selection of the reference category, we consider those with a low proportion of the outcome variable. The second comment is accepted as it is and the P-value of both the bivariate and multivariable analyses are incorporated after inserting extra columns.

**Abstract**

5. I do not feel that the first reviewer’s concerns about the abstract have been addressed. There needs to be a stronger rationale for the current study, and the conclusion needs to speak to the results. The current conclusion reads as though a mindfulness intervention for improving mental health was undertaken alongside improving household food security.

**Authors’ response:** the abstract, particularly the introduction and conclusion sections, is further revised. In the revised file, the rationale of the study is clearly stated and read as “Prenatal anxiety and depression are the major health problems all over the world. The negative sequela of prenatal comorbid anxiety and depression (CAD) has been suggested to be higher than those of anxiety or depression alone. CAD increases the odds of preterm birth, low birth weight, prolonged labor, operative deliveries, postpartum psychiatric disorders and long term cognitive impairment for the newborn. Despite its significant ill consequences, there is a dearth of studies in low-and middle-income countries; likewise, to the best of our knowledge, no study assessed the prevalence of CAD in Ethiopia. Hence, the purpose of this study was to assess CAD and associated factors among pregnant women in Arba Minch Zuria district, Gamo zone, southern Ethiopia.” Furthermore, the conclusion is reported based on the modifiable risk factors for comorbid anxiety and depression and read as “in general, one in every ten women faced CAD. Nutritional interventions, early identification and treatment of pregnancy-related illness and medical ailments, prenatal psychological screening and interventions are imperative to minimize the risk of CAD in pregnant women.”

Introduction

6. I do not feel that the prevalence of mental disorders in LMIC/ African antenatal populations was adequately discussed in the introduction, and noticed that there were African studies of mental health (including Ethiopian) which was not considered/cited. Authors cite Ethiopian studies in Line 66-68 – it would be interesting to know prevalence’s found in these studies for context and comparison.

I realise that the literature on comorbid depression and anxiety is sparse, but there are some studies which may be useful:

Sawyer A, Ayers S, Smith H. Pre- and postnatal psychological wellbeing in Africa: A systematic review. Journal of Affective Disorders 2010; 123(1–3): 17-29.

Baron EC, Hanlon C, Mall S, et al. Maternal mental health in primary care in five low- and middle-income countries: a situational analysis. BMC health services research 2016; 16: 53.

Redinger S, Pearson RM, Houle B, Norris SA, Rochat TJ. Antenatal depression and anxiety across pregnancy in urban South Africa. Journal of affective disorders 2020; 277: 296-305.

Biaggi A, Conroy S, Pawlby S, Pariante CM. Identifying the women at risk of antenatal anxiety and depression: A systematic review. Journal of Affective Disorders 2016; 191: 62-77.

(for medical condition comparison) Sowa NA, Cholera R, Pence BW, Gaynes BN. Perinatal depression in HIV-infected African women: a systematic review. *J Clin Psychiatry* 2015; **76**(10): 1385-96.

**Authors’ response**: first of all thank you for your critical review and very useful article suggestion. In the current appearance, the introduction section is almost changed after incorporating the recent accessible published evidence including your recommended articles. Regarding, studies conducted in Ethiopia, the studies merely address antenatal depression and the prevalence of depression is ranged from 11.8% to 31.2% and all the references are appropriately cited.

7. Line 45: Given the wide disparity in prevalences of depression and anxiety stated in the introduction, it would be helpful to have more information about the studies you cite when describing rates of comorbidity – for example, were participants from HIC/LMIC or low/high-risk pregnancies, etc. *There is an increasing trend in comorbid anxiety and depression (CAD); Falah et.al. reported that 9.5% of 25,592 participants had self-reported 46 antenatal anxiety and depressive symptoms*

**Authors’ response:** such misleading statements are critically reviewed and modified accordingly. Regarding the studies included in the meta-analysis conducted by Falah and coworkers most of the studies are from high-income countries; only three studies were included from Africa [1]. Besides, the introduction section is now written chronologically from the global to the local context.

Lines 50-55 explains the factors associated with “comorbid anxiety and depression” in the literature however only the bulk of cited literature is for either depression or anxiety. Might be useful to describe factors associated with one or the other in Africa/ Ethiopia specifically (as they are slightly different) and then tell the reader what is associated with comorbidity (this literature is sparse, might need to draw from HIC literature).

**Authors’ response:** Because of limited published evidence risk factors associated either with depression or anxiety were included. Almost all risk factors associated with depression/anxiety are also associated with comorbid anxiety and depression. In the revised file articles that assess the risk factors for comorbid anxiety and depression are cited.

**Methods**

8. When using the PHQ-9 as a measure of probable depression, a cut-off score of 10 is what has been shown in validation studies to represent a depression, including in antenatal populations. I know that a cutoff of >5 has been used in Ethiopia before, and I assume the authors have based their analysis on this however the reasoning given by the authors in the manuscript is confusing and makes it seem like prevalence’s are being under-estimated. I would reconsider this explanation. The same is true for the description of the GAD-7 cutoff and analysis.

*Line score of ≥5 was used as a cut-off point for possible antenatal depression [23] due to small/null observations under moderate and moderately severe and severe depression.*

**Authors’ response:** Primarily we plan to run multinomial logistic regression after categorizing the outcome variable as free of depression, mild, moderate, moderately severe and severe” based on WHO recommendation. However, when we run the frequency the observations were very low under moderate and moderately severe and almost null in the severe category. Then we decided to run a binary logistic regression by recoding the outcome variable to dummy variable as free of depression and with depression. Regarding the cut-of-point used as you mention, previously published studies in developed and some developing countries used a sore cut of point of 10. In our case, the PHQ Amharic version is validated in our countries context and a score of >=5 was considered as the optimal score for increased probability of major depressive disorder and used in Ethiopian studies [2,3]. Similarly, anxiety was categorized as mild, moderate and severe anxiety with cut points of 5, 10, and 15 respectively. But due to the small number of observations under moderate and severe anxiety, it was taken as a dummy variable with a cut-point of ≥ 5 [4]. In the revised, manuscript such inconsistencies and unnecessary statements were carefully addressed and amended.

**Results**

9. Lines 229 -231 Please state what the confounding factors in the model were?

**Authors’ response:** as it is reported in Table 3 both the unadjusted (Crude) OR and adjusted OR are reported. Those variables with a p-value of < 0.25 were candidates for multivariable analysis. After adjustment, some variables such as intimate partner violence, social support, and substance abuse were not significantly associated with comorbid anxiety and depression despite they were significantly associated in the bivariate analysis. Also, to assess whether the association is true for the rest of the variables we have to adjust all possible risk factors that have been associated with the outcome of interest. Let us explain it further: for example, household food insecurity is significantly associated with Comorbid anxiety and depression in the bivariate analysis (Crude OR = 5.32, 95% CI (3.13, 9.03) P < 0.001. The risk persists even after adjustment (adjusted OR= 3.51, 95% CI (1.85, 6.64) P < 0.001 for other known risk factors such as medical illness, marital status, a pregnancy danger sign, social support and intimate partner violence and wealth index. Hence, when we assess the association between household food insecurity and CAD the rest of the variables were considered as confounders. This explanation also works for the rest of the variables included in the final model.

Normal convention for reporting regression results: AOR 95% CI [CI-CI] p-value

**Authors’ response:** The P-value for the unadjusted and adjusted Odds ratio is presented in the final regression model. Adding the P-value in the text makes the sentence lengthy and boring for reading and we linked the text and readers can get the P-value from Table-3.

10. The fact that women in the highest socioeconomic bracket had higher odds of having comorbid depression and anxiety warrants a more detailed explanation/discussion – especially since women reporting food insecurity were at higher risk. It would be interesting to look at/ mention what the unadjusted results were for this variable to see if they were in the same direction, or if there was collinearity with the food insecurity variable in the multivariate regression.

**Authors’ response:** we asked this question many times at college and university level symposium presentations. We run the analysis many times and the association persists even after adjustment. The unadjusted OR with 95% CI and P-value for each variable are displayed in Table 3. Furthermore, as it is written under the statistical subsection of the method, multicollinearity was checked using standard error (SE); which is 1.14, 0.68 and 1.27 for household food insecurity, medium and high wealth quintile respectively. All these values are < 2, which confirms there is no collinearity.

Discussion

Lines 260 and 261 – consider referencing.

**Authors’ response:** the statement is appropriately cited.

11. Generally I echo the comments of the previous reviewer on the discussion section – that it is fairly written but would benefit from situating results within the LMIC then African then Ethiopian context.

**Authors’ response:** The discussion section, particularly the magnitude part is modified simultaneous with the introduction. In the revised file, additional references were accessed and cited appropriately by following the global to local chronological order.

12. Nice papers to look at when considering the results of food insecurity:

Trujillo J, Vieira MC, Lepsch J, et al. A systematic review of the associations between maternal nutritional biomarkers and depression and/or anxiety during pregnancy and postpartum. *Journal of Affective Disorders* 2018; **232**: 185-203.

*Abrahams Z, Lund C, Field S, Honikman S. Factors associated with household food insecurity and depression in pregnant South African women from a low socio-economic setting: a crosssectional study. Social psychiatry and psychiatric epidemiology 2018; 53(4): 363-72.*

**Authors’ response**: Thank you for suggesting these excellent articles; the second paper is significant for our paper and cited in the revised manuscript. The first one is interesting but it is not related to our study.

**Reviewer #4:** In my opinion, the authors have addressed all the comments and suggestions made by the reviewers and the manuscript could be published

**Authors’ response**: thank you for your suggestion

**Reviewer #5:** (No Response)

**Authors’ response**: I want to say thank you both the reviewers and academic editor for your critical review and suggestion; all the issues raised were carefully addressed. Still if there is any unclear or partially address concern we will amend further.

**References**

1. Falah-Hassani K, Shiri R, Dennis C-L (2017) The prevalence of antenatal and postnatal co-morbid anxiety and depression: a meta-analysis. Psychological medicine 47: 2041-2053.

2. Bitew T, Hanlon C, Kebede E, Medhin G, Fekadu A (2016) Antenatal depressive symptoms and maternal health care utilisation: a population-based study of pregnant women in Ethiopia. BMC Pregnancy Childbirth 16: 301.

3. Gelaye B, Williams MA, Lemma S, Deyessa N, Bahretibeb Y, et al. (2013) Validity of the patient health questionnaire-9 for depression screening and diagnosis in East Africa. Psychiatry research 210: 653-661.

4. Zhong Q-Y, Gelaye B, Zaslavsky AM, Fann JR, Rondon MB, et al. (2015) Diagnostic validity of the Generalized Anxiety Disorder-7 (GAD-7) among pregnant women. PloS one 10.
